# Absence of mating behaviors in the female dogs neonatally treated with estrogen and progesterone

**DOI:** 10.1590/1984-3143-AR2024-0080

**Published:** 2025-01-20

**Authors:** ChanJin Park, Kayla Tando, Sandra Soto-Heras, Sherry Zhou, Po-Ching Lin, CheMyong Ko

**Affiliations:** 1 Department of Comparative Biosciences, College of Veterinary Medicine, University of Illinois at Urbana-Champaign, Urbana, IL, USA; 2 Epivara, Inc., Champaign, IL, USA

**Keywords:** dog, estrogen, progesterone, neonatal, sexual behavior

## Abstract

This study aimed to develop a non-surgical method to neutralize reproduction in female dogs. Female Beagle puppies, aged 6 days, were treated with pellets designed to release estradiol benzoate (EB; 1.0 mg) and progesterone (P4; 5.0 mg) over approximately 3 weeks. Their estrous cycles were monitored from 6 to 34 months of age by examining their vulvas daily and measuring their serum P4 levels once a month. Vulvar edema and discharge, followed by a serum P4 level above 5 ng/ml, indicated the potential estrus. Each time a dog showed these signs, breeding was attempted by housing with a proven male Beagle. All the treated dogs displayed cyclic progesterone surges with 5 to 6-month-long anestrous intervals. Surprisingly, none exhibited sexual behaviors, and no mating occurred (i.e., no intromission and copulatory tie), resulting in no pups being born. This phenomenon was further explored in laboratory animals. Neonatal female rats were treated with microspheres containing smaller doses of the same steroids (0.3 mg EB + 3.0 mg P4) at 1 or 2 days old. At 3 months old, the rats were ovariectomized, chemically stimulated to exhibit estrus behaviors using a standard protocol and tested for receptivity to proven male rats. Untreated control rats showed normal receptivity (i.e., lordosis) and allowed males to mate. However, rats treated with EB+P4 did not exhibit lordosis or allow mating. These results indicate that the combined use of estrogen and progesterone could be an effective non-surgical method for inhibiting mating behavior and, consequently, neutralizing female dog reproduction.

## Introduction

Surgical sterilization has been a cornerstone of veterinary practice for numerous decades, providing an effective means of controlling animal population and mitigating health risks associated with sexual reproduction. However, recent advancements in veterinary medicine and the growing emphasis on less invasive procedures have highlighted the need to explore non-surgical sterilization methods (Ladd et al., 1994; Asa, 2018). Moreover, cost-effective methods for sterilizing animals are urgently needed to address the overpopulation challenges faced by animal shelters.

Recently, Kisspeptin neurons in the hypothalamus have emerged as a potential target for inducing infertility in animals. These neurons secrete the master reproductive hormone, Kisspeptin (KISS1) (Harter et al., 2018; Uenoyama et al., 2018), which controls the release of gonadotropin-releasing hormone (GnRH) (Smith et al., 2018). Ablation of either the *Kiss1* or *Kiss1r* gene resulted in hypogonadism and permanent infertility in rats (d'Anglemont de Tassigny et al., 2007; Novaira et al., 2014). In our previous study, we demonstrated that a single injection of estradiol benzoate (EB) via a slow-release method effectively inhibited the development of Kisspeptin neurons in rats, leading to permanent infertility (Park et al., 2023). Furthermore, we showed that a similar treatment also reduced hypothalamic *KISS1* mRNA expression in female dogs (Park et al., 2023). These results indicate that non-invasive sterilization of animals can be achieved by administering estrogen during the neonatal period.

Additionally, neonatal treatment with estrogen ‘reprograms’ sexual behaviors in females. Adult female rats that received EB after birth did not exhibit lordosis responses during breeding trials, despite maintaining vaginal cyclicity (Whalen and Nadler, 1965; Henley et al., 2009). The effect of neonatal estradiol on sexual behavior is likely central. Female and male brains present significant anatomical and functional differences, including neuron numbers, synapses, and gene expression, that are established after birth during a sensitive period of reproductive development (McCarthy, 2009). This developmental programming is driven by high levels of circulating testosterone in males, which is locally converted into estradiol (E2) in the brain by the aromatase enzyme and can be replicated in females by exogenous estrogen exposure. For instance, treatment of neonatal female rats with exogenous estradiol increased the numbers of neurons in the sexually dimorphic nucleus of the preotic area (SDN-POA) (Davis et al., 1996).

While it is known that neonatal exposure to estrogen disrupts the development of the female reproductive axis and reprograms female reproductive behavior, exogenous estrogen may have other systemic effects in organs expressing estrogen receptors (ESRs). Adverse effects have been described with the use of some estrogenic compounds at specific doses. For instance, exposure to 17alpha-ethynylestradiol, a potent synthetic derivative of estradiol, during the neonatal period induced mammary gland hyperplasia (Shiorta et al., 2012; Shirota et al., 2015). On the other hand, progesterone (P4) is known to downregulate the expression of ESRs in the mammary glands, thereby inhibiting estrogen-induced epithelial cell proliferation (Mauvais-Jarvis et al., 1986; Foidart et al., 1998). Similarly, P4 blocks the growth-promoting effects of estrogen in the uterine endometrium by reducing cytoplasmic estrogen receptor levels (Anderson et al., 1977). In the pituitary, P4 antagonizes the permissive action of estrogens on the apoptosis of lactotrophs and somatotrophs (Candolfi et al., 2005).

Administering EB and P4 simultaneously could potentially reduce the off-target effects due to P4 counteracting estrogen's actions in various organs. However, this counteraction might also diminish the effectiveness of estrogen in inducing infertility. Therefore, this study aimed to test whether co-administration of P4 with estrogen will still effectively neutralize reproduction in female animals.

## Methods

### Ethics statement

This study was carried out in accordance with the recommendations in the Guide for the Care and Use of Laboratory Animals of the National Institutes of Health and ARRIVE guideline (Kilkenny et al., 2010). The dog study protocol was reviewed and approved by Institutional Animal Care and Use Committee of Ridglan Farms, AAALAC accredited research facility (Protocol: IN-100-006), and all efforts were made to minimize animal suffering. The rats experiment was approved by the University of Illinois Animal Care and Use Committee (Protocol: 23003).

### Animals

Female Beagles (*Canis lupus familiaris*) were purchased and housed at a Class R research facility, Ridglan Farm (Ridglan Inc., Blue Mounds, WI). Dogs were maintained under controlled lighting (12 h light/12 h dark). Newborn puppies were housed with their mothers and received their mother’s milk until weaning. The pups were fed a commercially available feed and water was available ad libitum from automatic waterers. Sprague Dawley rats were purchased from Charles River Laboratory, bred at the University of Illinois Division of Animal Resources, and maintained under controlled lighting (12 h light/12 h dark) with continuous access to food and water. Rats were acclimatized for one week prior to beginning the experimental procedures. Breeder pairs were housed together for 14 days, with pregnant females then being housed individually, and pups were weaned at 21 days of age.

### Experimental design

EB-loaded pellets (EB-pellets) and P4-loaded pellets (P4-pellets) were purchased from Innovative Research of America (Cat. E-281; Sarasota, FL). EB-loaded PLGA microspheres (EBMS; Lot 6028-119, 17.1% EB) and P4-loaded PLGA microspheres (P4MS; Lot 6028-126, 17.1% EB) were synthesized by SpineThera (Minneapolis, MN) and reconstituted with 50 µL PBS solution (pH 7.4) prior to injection by syringe.

Female Beagles were implanted with an EB-pellet containing 1.0 mg EB and a P4-pellet containing 5.0 mg P4 (EB 2.5 mg/kg BW and P4 12.5 mg/kg BW; n=2) on postnatal day (PND) 6. These doses were determined based on our previous rodent study results (Park et al., 2023), using the inter-species dose conversion formula (Nair and Jacob, 2016), and considering the estrogen-to-progesterone ratio in the blood of rats and dogs (Maier and Herman, 2001). The implants were placed subcutaneously in the nape. For the measurement of monthly serum P4 levels, peripheral blood was collected at each time point by hind limb vessel venipuncture. The blood was centrifuged for 10 minutes, and serum was collected. A P4 ELISA kit (Cat. EIA1561, DRG Diagnostic) with a reportable range of 0-42 ng/mL was used to measure circulating P4. The intra- and inter-assay coefficients of variability were less than 10%. Monthly P4 measurements and daily physical observation of vulvar edema and discharge for each dog were conducted from 6 to 34 months of age. The first observation of vulvar serosanguineous discharge was designated as the potential onset of estrus. Four to five days after this observation, the females were housed with fertility-proven male Beagles to observe mating behavior. Regardless of successful mating (i.e., male intromission and formation of copulatory tie), each female was allowed to mate with three different male Beagles, with a two-day interval between each mating attempt. Therefore, a total of three mating trials, each lasting 1 hour, were performed for each female displaying serosanguineous discharge. Although the current study did not include control females, the same males were bred with four females not involved in the study but were approximately the same age as the study dogs (within ±3 weeks). Results from this non-study breeding was used to verify that the males were fertile and the testing environment was favorable for breeding.

**Figure 1:**
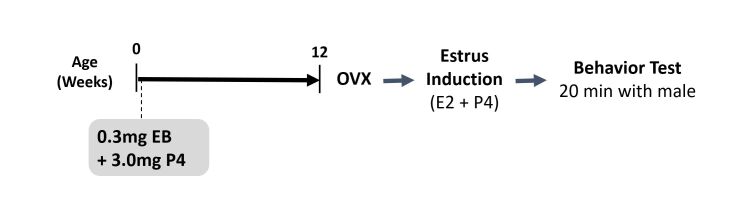
Experimental design of the mating behavior test in rats neonatally treated with estradiol benzoate. At 12 weeks, rats were ovariectomized and primed with estradiol (E2) and progesterone (P4). Then, rats were paired with a male of proven fertility and their behavior was recorded for 20 minutes.

A trial was conducted in rats to determine if co-treatment of EB and P4 to neonates would induce mating behaviors similar to those observed in treated dogs (Figure 1). The female rats were treated with vehicle (control group; n=7) or with microspheres containing 0.3 mg EB and 3.0 mg P4 (EB 40 mg/kg BW and P4 400 mg/kg BW; n=5) on PND1. When they reached post-pubertal age, their mating behavior was determined following an established protocol (Edwards and Pfeifle, 1983). Briefly, at 2.5 months of age, they were ovariectomized (OVX) and given at least 10 days of recovery. At 3 months of age, the OVX rats were injected subcutaneously with EB (50 ug/kg), followed by an injection of P4 (2.5 mg/kg) 40 to 48 hours after the EB injection, to induce their estrus response. This protocol ensures that any differences detected on mating behavior are not due to differences in concentrations of blood steroids (Bonthuis et al., 2011). Four hours after the P4 injection, their sexual behavior was tested in a quiet dark room illuminated with dim red lights. Each test subject was put in a large cage with a proven fertile male rat for 20 minutes. The number of the male rat mounting attempts and the female’s lordosis posture (i.e., head elevation with tail and back in dorsiflexion) were recorded. The testing result was presented as the lordosis quotient (i.e., the number of lordosis postures/number of mounts).

### Statistical analysis

Data analyses were performed using Microsoft Excel, GraphPad Prism (v9.3.1), and SPSS (v18.0). Continuous data were analyzed for normal distribution by a Shapiro-Wilk test. All normally distributed continuous data were analyzed with parametric tests (Student’s t-test). Data are graphically presented as the mean ±standard deviation (SD), and statistically significant differences were accepted when *P*-value was lower than 0.05.

## Results

**Figure 2:**
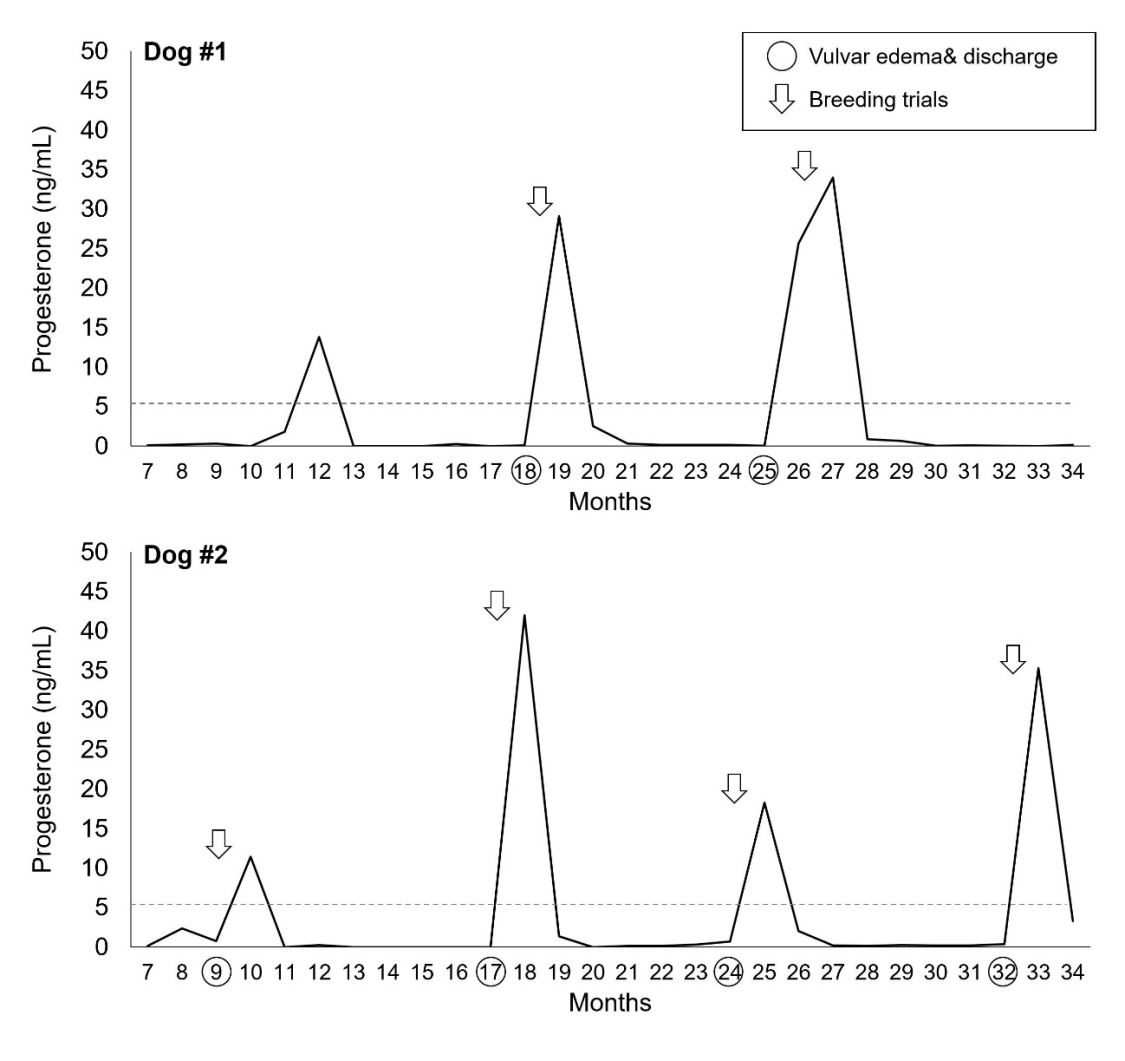
The patterns of progesterone increase exhibited by female Beagle dogs neonatally treated with estradiol benzoate and progesterone. Circles indicate the months of age when vulvar edema and discharge were observed. Arrows indicate the ages when breeding trials were performed in relation to the timing of observing vulvar edema/discharge and increased serum progesterone levels.

Two dogs treated with 1.0 mg EB and 5.0 mg P4 exhibited periodic spikes in serum progesterone levels, with 5-6 month-long anestrous intervals (Figure 2). They exhibited three and four P4 peaks (>5 ng/mL), respectively, over a 28-month observation period from 6 months of age, each preceded by vulvar discharge, except for the first P4 peak in Dog #1. Four to five days after observing vulvar discharge, a series of breeding trials were conducted over a week. In each trial, each female was paired with a different male, resulting in testing with a total of three different male partners (see the Materials and Methods for detail). During these trials, neither dog was receptive to the approach of male dogs, exhibited rejection behaviors towards mating attempts, and had no standing estrus; therefore, male intromission did not take place. In contrast, four female dogs that were not included in the study, but housed under the same conditions in the same facility showed normal mating behaviors (i.e., observed formation of copulatory tie), became pregnant, and successfully produced litters. These results suggest that while the neonatal treatment of EB and P4 did not affect the patterns of P4 surges, it resulted in the absence of mating behaviors.

**Figure 3:**
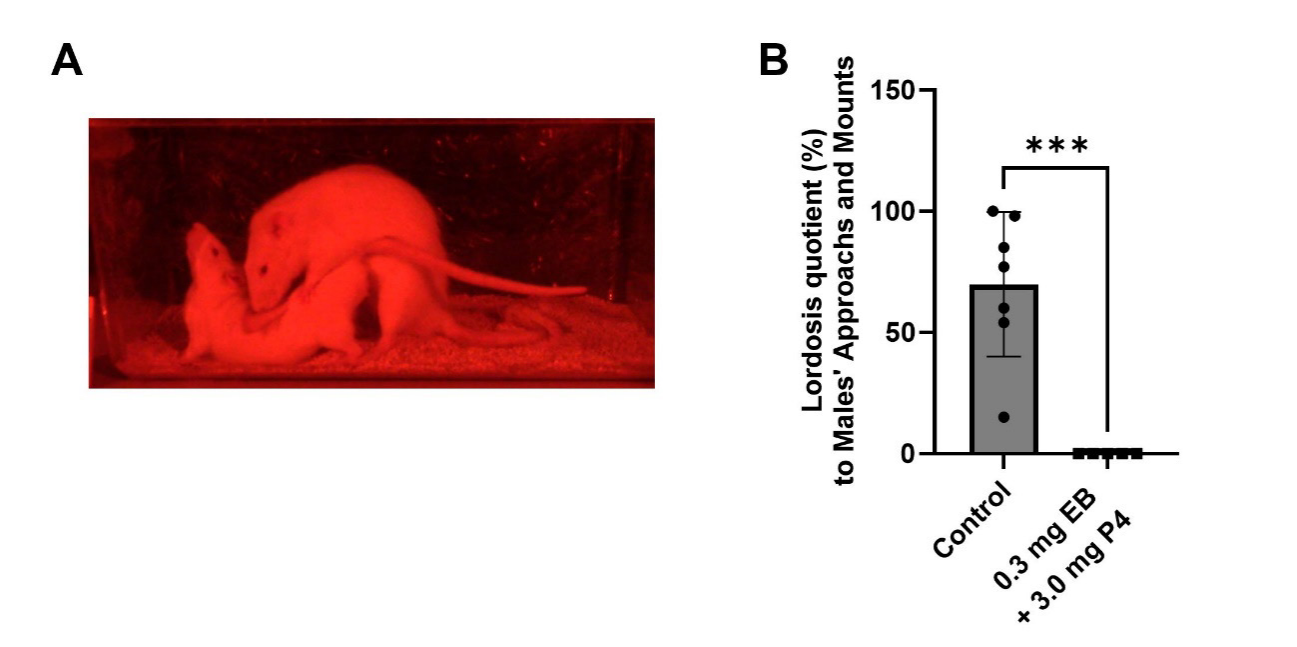
Lack of mating behavior exhibited by female rats neonatally treated with estradiol benzoate and progesterone when paired with a male of proven fertility. (A) Representative lordosis/mating posture displayed by a control female rat and a proven male rat during the trial. (B) Lordosis quotient female rats to male’s mounting attempts during the mating behavior trial (n=7 in the control group, n=5 in the EB+P4 group). Error bars, SD; ***, *P*-value < 0.005 (Student’s t-test).

In a 20-minute mating behavior test, the lordosis quotient in control rats was 69.9±29.8% in response to male mounting attempts (Figure 3). In contrast, rats treated with 0.3 mg EB and 3.0 mg P4 exhibited a 0% frequency of lordosis responses (*P*=0.004), mirroring the absence of mating behavior observed in the treated dogs.

## Discussion

Previous studies have shown that neonatal EB treatment induces infertility in female rats, with concomitant and irreversible inhibition of Kisspeptin expression (Minabe et al., 2017; Park et al., 2023). In the present study, female dogs co-treated with EB and P4 exhibited cyclic vulvar edema and discharge and cyclic elevation of serum P4 levels, but presented complete absence of mating behaviors.

The question remains whether P4 co-treatment counteracts the effect of EB on Kisspeptin neurons and possibly other estrogen actions in these dogs. While conclusive answers cannot be drawn from this study, the established roles of P4 in antagonizing estrogen actions in the uterus and mammary glands by decreasing ESR expression point to such possibility (Anderson et al., 1977; Mauvais-Jarvis et al., 1986; Foidart et al., 1998). A potentially similar effect of P4 on ESRs expression in the neonatal hypothalamus may hinder the action of estrogen on the anteroventral periventricular nucleus (AVPV) Kisspeptin neurons, consequently keeping the machinery for regulating the hypothalamic-pituitary-gonadal axis intact or less affected. It would be worth exploring whether using a selective estrogen receptor agonist or modulator could achieve similar efficacy in the hypothalamus while minimizing effects on other estrogen-responsive organs.

The most interesting finding from this study is that while the dogs treated with EB+P4 exhibited cyclic vulvar discharge and progesterone surges, they did not display mating behavior towards mature and fertility-proven male dogs. Further, this finding was corroborated in an independent rat study. In this study, before the behavior test, both control and treated rats were ovariectomized and hormonally primed. The lack of sexual behavior in treated rats, despite having equivalent circulating steroids to control rats, indicates that the effect on behavior is likely central. Indeed, previous studies showed that neonatal exposure to exogenous estrogens alters the differentiation of brain areas controlling reproductive behavior (McCarthy, 2020) and, consequently, ‘reprograms’ the female’s sexual behavior in adulthood (Bakker and Baum, 2008; McCarthy, 2008; Henley et al., 2009). Although the effect of progesterone is not fully understood, there is evidence that it may also play a role in brain sexual development. Notably, during the neonatal period, progesterone receptors (PGRs) are expressed in neurons of various regions, including the medial preoptic area (MPOA), with different expression levels in males and females (Quadros et al., 2002a; Quadros et al., 2002c; Gonzales et al., 2012). Moreover, neonatal treatment of a PGR antagonist to female rats (RU486) attenuated the effects of testosterone on increasing the volume of the SDN-POA (Quadros et al., 2002b).

In summary, this study demonstrates that neonatal treatment of a combination of estrogen and progesterone to female puppies results in the absence of sexual receptivity post-pubertally. Future research should focus on identifying the optimal timing and safe dosage needed to advance this approach as a non-surgical sterilization method for female dogs.

## Conclusion

While the mechanism behind the inhibition of mating behavior caused by neonatal treatment with EB and P4 has not been fully elucidated, the results of the present study indicate that this approach may open an avenue for developing a novel method of inducing infertility in companion animals without the surgical removal of their gonads.
